# 4,4-Dimethyl-3,4-dihydro­pyrido[2′,3′:3,4]pyrazolo­[1,5-*a*][1,3,5]triazin-2-amine ethanol monosolvate^1^

**DOI:** 10.1107/S160053681005097X

**Published:** 2010-12-11

**Authors:** Anton V. Dolzhenko, Geok Kheng Tan, Anna V. Dolzhenko, Lip Lin Koh, Wai Keung Chui

**Affiliations:** a School of Pharmacy, Faculty of Health Sciences, Curtin University of Technology, GPO Box U1987, Perth 6845, Western Australia, Australia; b Department of Chemistry, Faculty of Science, National University of Singapore, 3 Science Drive 3, Singapore 117543, Singapore; c Perm State Pharmaceutical Academy, 2 Polevaya Street, Perm 614990, Russian Federation; d Department of Pharmacy, Faculty of Science, National University of Singapore, 18 Science Drive 4, Singapore 117543, Singapore

## Abstract

In the title compound, C_10_H_12_N_6_·C_2_H_5_OH, the planarity of the heterocyclic system is slightly distorted at the triazine ring (r.m.s. deviation = 0.1191 Å), which adopts a conformation best described as inter­mediate between a flattened twisted boat and a half-boat with the tertiary C*sp* ^3^ atom at the bow. In the crystal, mol­ecules form centrosymmetric dimers connected by N⋯H—O and O⋯H—N hydrogen bonds between the amino group H atom, the ethanol solvent mol­ecule and the triazine N atom, making an *R*_4_^4^(12) graph-set motif. The other H atom of the amino group and the H atom on the endocyclic N atom form N⋯H—N hydrogen bonds with the N atoms of the pyrazole and pyridine rings, respectively, linking the mol­ecules into *C*(7)*C*(7) chains with the *R*_2_^2^(8) binary graph-set motif running along [010].

## Related literature

For a review on the synthesis and biological activity of pyrazolo­[1,5-*a*]triazines, see: Dolzhenko *et al.* (2008 ▶). For the synthesis, crystal structure studies and biological activity of related fused *gem*-dimethyl-substituted amino-1,3,5-triazines, see: Dolzhenko *et al.* (2007*a* ▶,*b* ▶, 2009 ▶), Toyoda *et al.* (1997 ▶). For graph-set analysis of hydrogen bonding, see: Bernstein *et al.* (1995 ▶). For a related structure, see: Sachdeva *et al.* (2010 ▶).
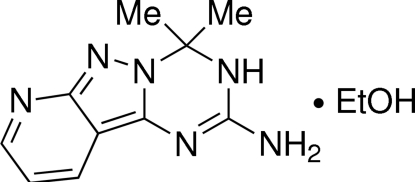


## Experimental

### 

#### Crystal data

C_10_H_12_N_6_·C_2_H_6_O
*M* *_r_* = 262.32
Monoclinic, 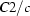

*a* = 12.1250 (19) Å
*b* = 13.913 (2) Å
*c* = 16.598 (3) Å
β = 101.683 (4)°
*V* = 2742.0 (7) Å^3^
*Z* = 8
Mo *K*α radiation
μ = 0.09 mm^−1^
*T* = 100 K
0.60 × 0.38 × 0.10 mm

#### Data collection

Bruker SMART APEX CCD diffractometer
Absorption correction: multi-scan (*SADABS*; Sheldrick, 2001 ▶) *T*_min_ = 0.950, *T*_max_ = 0.991
9471 measured reflections
3129 independent reflections
2657 reflections with *I* > 2σ(*I*)
*R*_int_ = 0.030

#### Refinement

*R*[*F* ^2^ > 2σ(*F* ^2^)] = 0.050
*wR*(*F* ^2^) = 0.133
*S* = 1.06
3129 reflections
209 parameters
38 restraints
H atoms treated by a mixture of independent and constrained refinement
Δρ_max_ = 0.37 e Å^−3^
Δρ_min_ = −0.21 e Å^−3^

### 

Data collection: *SMART* (Bruker, 2001 ▶); cell refinement: *SAINT* (Bruker, 2001 ▶); data reduction: *SAINT*; program(s) used to solve structure: *SHELXS97* (Sheldrick, 2008 ▶); program(s) used to refine structure: *SHELXL97* (Sheldrick, 2008 ▶); molecular graphics: *SHELXTL* (Sheldrick, 2008 ▶); software used to prepare material for publication: *SHELXTL*.

## Supplementary Material

Crystal structure: contains datablocks I, global. DOI: 10.1107/S160053681005097X/hg2760sup1.cif

Structure factors: contains datablocks I. DOI: 10.1107/S160053681005097X/hg2760Isup2.hkl

Additional supplementary materials:  crystallographic information; 3D view; checkCIF report

## Figures and Tables

**Table 1:** Hydrogen-bond geometry (Å, °)

*D*—H⋯*A*	*D*—H	H⋯*A*	*D*⋯*A*	*D*—H⋯*A*
N5—H5⋯N1^i^	0.86 (2)	2.16 (2)	3.0077 (18)	169.5 (18)
N6—H6*B*⋯N2^i^	0.90 (2)	2.08 (2)	2.9754 (18)	171.5 (18)
N6—H6*A*⋯O1*S*^ii^	0.85 (2)	2.03 (2)	2.8540 (18)	163.2 (18)
O1*S*—H1*S*⋯N4	0.87 (2)	1.93 (2)	2.7943 (17)	170 (2)

Symmetry codes: (i) 
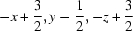
; (ii) 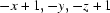.

**Fig. 1.**
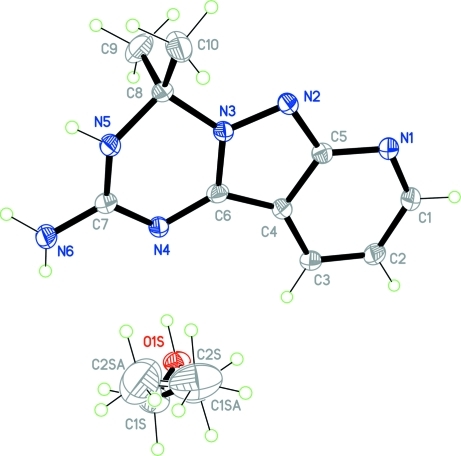
The molecular structure of 4,4-dimethyl-3,4-dihydropyrido[2',3':3,4]pyrazolo[1,5-a][1,3,5]triazin-2-amine ethanol solvate showing the atomic numbering scheme. Displacement ellipsoids are drawn at the 50% probability level.

**Fig. 2.**
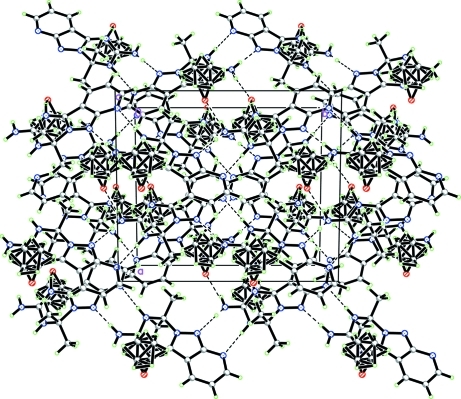
Crystal packing in the cell (view along axis c).

**Fig. 3.**
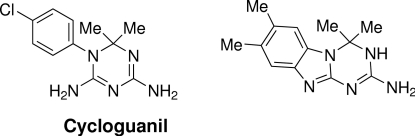
Dihydrofolate reductase inhibitors with dihydro-1,3,5-triazine ring.

**Fig. 4.**
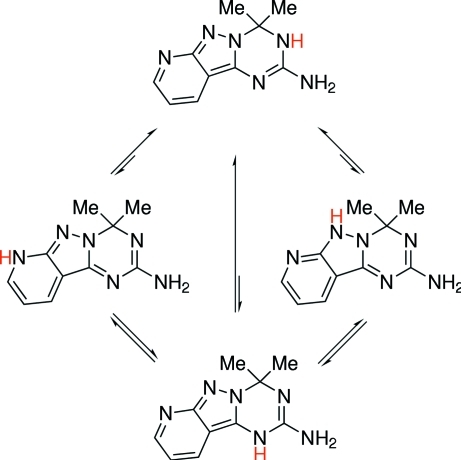
Annular tautomerism in 4,4-dimethyl-3,4-dihydropyrido[2',3':3,4]pyrazolo[1,5-a][1,3,5]triazin-2-amine.

## supplementary crystallographic information

### Comment

The pyrazolo[1,5-*a*]triazine heterocyclic system has been recognized as a template for the construction of new potential therapeutic agents (Dolzhenko *et al.*, 2008). However data on pyrido[2',3':3,4]pyrazolo[1,5-*a*][1,3,5]triazines are limited and no details on their structure are available. Herein, we report molecular and crystal structure of 4,4-dimethyl-3,4-dihydropyrido[2',3':3,4]pyrazolo[1,5-*a*][1,3,5]triazin-2-amine, which crystallizes with a ethanol molecule to give the title compound, C_10_H_12_N_6_.C_2_H_5_OH (Fig. 1 & 2). This molecule has a close structural resemblance with some known dihydrofolate reductase inhibitors such as antimalarial drug cycloguanil and its fused analogue (Toyoda *et al.*, 1997) (Fig. 3). Due to annular tautomerism, four tautomeric forms are theoretically possible for the compound (Fig. 4). Similarly to the previously reported (Dolzhenko *et al.*, 2007*b*) related fused *gem*-dimethyl substituted amino-1,3,5-triazine, the compound exists in the crystal as a tautomer with the labile hydrogen atom located at the triazine nitrogen atom adjacent to the *sp*^3^ hybridized carbon atom.

The heterocyclic system is nearly planar (with r.m.s. deviation of 0.1191 Å) with a distortion at the triazine ring. The triazine ring adopts the conformation best described as an intermediate between a flatten twist boat and a half-boat with atoms C-8 and N-4 at the bow and the stern. The angle between the geminal flagpole and bowsprit methyl groups is 112.40 (14)°. The N4—C7, N5—C7 and N6—C7 bond distances are similar suggesting guanidine-like electron delocalization in the N4—N6/C7 fragment of the molecule.

In the crystal, the molecules form centrosymmetric dimers connected by the N···HO and O···HN hydrogen bonds between the amino group N6—H6A, ethanol molecule and the triazine N4 atom making a *R*_4_^4^(12) graph-set motif (Bernstein *et al.*, 1995). Another hydrogen atom of the amino group N6—H6B and the hydrogen atom at the endocyclic N5 atom act as hydrogen donors forming N···HN contacts with the pyrazole and pyridine N2 and N1 atoms, respectively. They arrange molecules into the running along a [010] axis *C*(7)*C*(7) chains with the *R*_2_^2^(8) binary graph-set motif.

### Experimental

4,4-Dimethyl-3,4-dihydropyrido[2',3':3,4]pyrazolo[1,5-*a*][1,3,5]triazin-2-amine was prepared by the cyclocondensation of pyrazolo[3,4-*b*]pyridin-3-guanidine with acetone similarly to the previously described methods (Dolzhenko *et al.*, 2007*a*; Dolzhenko *et al.*, 2009). 1*H*-Pyrazolo[3,4-*b*]pyridin-3-guanidine (0.88 g, 5.0 mmol) and piperidine (0.30 ml, 3.0 mmol) were heated in acetone (30 ml) under reflux for 10 h. After cooling, the product was filtered and washed with acetone. Yield: 0.89 g (82%). The crystals suitable for crystallographic analysis were grown by recrystallization from ethanol. m.p. 559 K.

### Refinement

All C-bound H atoms were positioned geometrically and included in the refinement in riding-motion approximation [0.95 Å for CH of pyridine ring, 0.98 Å for methyl groups, and 0.99 Å for methylenic protons; *U*_iso_(H) = 1.2*U*_eq_(C_py_), *U*_iso_(H) = 1.5*U*_eq_(C_Me_) and *U*_iso_(H)= 1.2*U*_eq_(C_methylenic_)] while the N-bound H atoms were located in a difference map and refined freely. The ethyl group of ethanol molecule was disordered into two positions with occupancy ratio of 78:22.

### Crystal data

**Table:**

C_10_H_12_N_6_·C_2_H_6_O	*F*(000) = 1120
*M**_r_* = 262.32	*D*_x_ = 1.271 Mg m^−^^3^
Monoclinic, *C*2/*c*	Melting point: 559 K
Hall symbol: -C 2yc	Mo *K*α radiation, λ = 0.71073 Å
*a* = 12.1250 (19) Å	Cell parameters from 782 reflections
*b* = 13.913 (2) Å	θ = 2.7–27.3°
*c* = 16.598 (3) Å	µ = 0.09 mm^−^^1^
β = 101.683 (4)°	*T* = 100 K
*V* = 2742.0 (7) Å^3^	Plate, yellow
*Z* = 8	0.60 × 0.38 × 0.10 mm

### Data collection

**Table:**

Bruker SMART APEX CCD diffractometer	3129 independent reflections
Radiation source: fine-focus sealed tube	2657 reflections with *I* > 2σ(*I*)
graphite	*R*_int_ = 0.030
φ and ω scans	θ_max_ = 27.5°, θ_min_ = 2.3°
Absorption correction: multi-scan (*SADABS*; Sheldrick, 2001)	*h* = −15→14
*T*_min_ = 0.950, *T*_max_ = 0.991	*k* = −18→17
9471 measured reflections	*l* = −18→21

### Refinement

**Table:**

Refinement on *F*^2^	Primary atom site location: structure-invariant direct methods
Least-squares matrix: full	Secondary atom site location: difference Fourier map
*R*[*F*^2^ > 2σ(*F*^2^)] = 0.050	Hydrogen site location: inferred from neighbouring sites
*wR*(*F*^2^) = 0.133	H atoms treated by a mixture of independent and constrained refinement
*S* = 1.06	*w* = 1/[σ^2^(*F*_o_^2^) + (0.0709*P*)^2^ + 1.8445*P*] where *P* = (*F*_o_^2^ + 2*F*_c_^2^)/3
3129 reflections	(Δ/σ)_max_ = 0.001
209 parameters	Δρ_max_ = 0.37 e Å^−^^3^
38 restraints	Δρ_min_ = −0.21 e Å^−^^3^

### Special details

**Table:**

Geometry. All e.s.d.'s (except the e.s.d. in the dihedral angle between two l.s. planes) are estimated using the full covariance matrix. The cell e.s.d.'s are taken into account individually in the estimation of e.s.d.'s in distances, angles and torsion angles; correlations between e.s.d.'s in cell parameters are only used when they are defined by crystal symmetry. An approximate (isotropic) treatment of cell e.s.d.'s is used for estimating e.s.d.'s involving l.s. planes.
Refinement. Refinement of *F*^2^ against ALL reflections. The weighted *R*-factor *wR* and goodness of fit *S* are based on *F*^2^, conventional *R*-factors *R* are based on *F*, with *F* set to zero for negative *F*^2^. The threshold expression of *F*^2^ > σ(*F*^2^) is used only for calculating *R*-factors(gt) *etc*. and is not relevant to the choice of reflections for refinement. *R*-factors based on *F*^2^ are statistically about twice as large as those based on *F*, and *R*- factors based on ALL data will be even larger.

### Fractional atomic coordinates and isotropic or equivalent isotropic displacement parameters (Å^2^)

**Table:**

	*x*	*y*	*z*	*U*_iso_*/*U*_eq_	Occ. (<1)
O1S	0.49619 (10)	0.11324 (8)	0.46397 (7)	0.0285 (3)	
H1S	0.5246 (18)	0.1117 (15)	0.5165 (14)	0.039 (6)*	
C1S	0.5838 (2)	0.0860 (2)	0.42339 (15)	0.0384 (7)	0.781 (6)
H1SA	0.5526	0.0769	0.3640	0.046*	0.781 (6)
H1SB	0.6169	0.0243	0.4461	0.046*	0.781 (6)
C2S	0.6736 (3)	0.1618 (3)	0.4346 (2)	0.0648 (11)	0.781 (6)
H2SA	0.7335	0.1420	0.4063	0.097*	0.781 (6)
H2SB	0.7051	0.1701	0.4934	0.097*	0.781 (6)
H2SC	0.6410	0.2226	0.4114	0.097*	0.781 (6)
C1SA	0.5898 (9)	0.1503 (9)	0.4290 (6)	0.044 (2)	0.219 (6)
H1SC	0.5651	0.1572	0.3687	0.053*	0.219 (6)
H1SD	0.6120	0.2146	0.4523	0.053*	0.219 (6)
C2SA	0.6822 (14)	0.0883 (12)	0.4464 (9)	0.077 (4)	0.219 (6)
H2SD	0.7437	0.1142	0.4227	0.116*	0.219 (6)
H2SE	0.6605	0.0250	0.4226	0.116*	0.219 (6)
H2SF	0.7072	0.0823	0.5062	0.116*	0.219 (6)
N1	0.56023 (10)	0.47488 (8)	0.67409 (7)	0.0203 (3)	
N2	0.67666 (10)	0.34962 (9)	0.74160 (7)	0.0205 (3)	
N3	0.68205 (10)	0.25362 (8)	0.72312 (7)	0.0187 (3)	
N4	0.59745 (10)	0.13025 (8)	0.63025 (7)	0.0208 (3)	
N5	0.77009 (11)	0.10579 (9)	0.72296 (8)	0.0220 (3)	
H5	0.8212 (17)	0.0662 (15)	0.7465 (12)	0.034 (5)*	
N6	0.68817 (13)	−0.01616 (10)	0.63779 (9)	0.0302 (3)	
H6A	0.6334 (17)	−0.0354 (14)	0.6007 (12)	0.028 (5)*	
H6B	0.7326 (17)	−0.0588 (15)	0.6701 (12)	0.037 (5)*	
C1	0.47528 (13)	0.49206 (10)	0.61227 (9)	0.0226 (3)	
H1	0.4479	0.5562	0.6059	0.027*	
C2	0.42132 (13)	0.42321 (11)	0.55453 (9)	0.0235 (3)	
H2	0.3604	0.4420	0.5119	0.028*	
C3	0.45708 (12)	0.32946 (11)	0.56021 (9)	0.0211 (3)	
H3	0.4242	0.2824	0.5212	0.025*	
C4	0.54476 (12)	0.30661 (10)	0.62667 (8)	0.0182 (3)	
C5	0.59298 (12)	0.38088 (10)	0.68156 (8)	0.0180 (3)	
C6	0.60627 (12)	0.22357 (10)	0.65668 (8)	0.0189 (3)	
C7	0.68259 (13)	0.07377 (11)	0.66450 (9)	0.0220 (3)	
C8	0.75702 (12)	0.18664 (10)	0.77658 (8)	0.0210 (3)	
C9	0.70180 (17)	0.15481 (14)	0.84683 (10)	0.0384 (4)	
H9A	0.6953	0.2100	0.8823	0.058*	
H9B	0.7480	0.1049	0.8791	0.058*	
H9C	0.6266	0.1291	0.8243	0.058*	
C10	0.87144 (15)	0.23234 (12)	0.80702 (12)	0.0363 (4)	
H10A	0.8983	0.2606	0.7604	0.055*	
H10B	0.9249	0.1832	0.8332	0.055*	
H10C	0.8651	0.2827	0.8471	0.055*	

### Atomic displacement parameters (Å^2^)

**Table:**

	*U*^11^	*U*^22^	*U*^33^	*U*^12^	*U*^13^	*U*^23^
O1S	0.0298 (6)	0.0327 (6)	0.0198 (6)	0.0020 (5)	−0.0023 (5)	−0.0027 (4)
C1S	0.0441 (15)	0.0418 (17)	0.0304 (12)	0.0038 (12)	0.0104 (10)	−0.0026 (11)
C2S	0.050 (2)	0.101 (3)	0.0473 (17)	−0.0189 (19)	0.0193 (14)	0.0019 (18)
C1SA	0.044 (5)	0.049 (6)	0.040 (4)	−0.019 (4)	0.008 (4)	0.007 (4)
C2SA	0.089 (8)	0.077 (8)	0.069 (7)	0.018 (6)	0.021 (6)	0.022 (6)
N1	0.0230 (6)	0.0168 (6)	0.0203 (6)	−0.0004 (5)	0.0025 (5)	0.0008 (5)
N2	0.0230 (6)	0.0159 (6)	0.0205 (6)	0.0006 (5)	−0.0001 (5)	−0.0021 (5)
N3	0.0195 (6)	0.0158 (6)	0.0184 (6)	0.0010 (4)	−0.0016 (5)	−0.0014 (4)
N4	0.0233 (6)	0.0164 (6)	0.0192 (6)	0.0016 (5)	−0.0042 (5)	−0.0017 (4)
N5	0.0227 (6)	0.0180 (6)	0.0217 (6)	0.0050 (5)	−0.0042 (5)	−0.0022 (5)
N6	0.0356 (8)	0.0196 (7)	0.0273 (7)	0.0063 (6)	−0.0128 (6)	−0.0040 (5)
C1	0.0255 (8)	0.0178 (7)	0.0235 (7)	0.0024 (6)	0.0027 (6)	0.0018 (6)
C2	0.0234 (7)	0.0232 (7)	0.0209 (7)	0.0015 (6)	−0.0028 (6)	0.0024 (6)
C3	0.0223 (7)	0.0213 (7)	0.0177 (7)	−0.0012 (5)	−0.0002 (5)	−0.0009 (5)
C4	0.0190 (7)	0.0170 (7)	0.0183 (7)	−0.0008 (5)	0.0029 (5)	−0.0004 (5)
C5	0.0173 (6)	0.0190 (7)	0.0177 (6)	−0.0011 (5)	0.0033 (5)	0.0001 (5)
C6	0.0185 (7)	0.0209 (7)	0.0158 (6)	−0.0005 (5)	0.0003 (5)	0.0001 (5)
C7	0.0254 (7)	0.0201 (7)	0.0182 (7)	0.0013 (6)	−0.0011 (6)	−0.0004 (5)
C8	0.0226 (7)	0.0194 (7)	0.0179 (7)	0.0046 (5)	−0.0031 (6)	−0.0015 (5)
C9	0.0475 (11)	0.0425 (10)	0.0257 (8)	0.0202 (8)	0.0088 (8)	0.0115 (7)
C10	0.0286 (9)	0.0244 (8)	0.0466 (10)	0.0042 (7)	−0.0146 (8)	−0.0081 (7)

### Geometric parameters (Å, °)

**Table:**

O1S—C1S	1.420 (3)	N5—C7	1.3596 (18)
O1S—C1SA	1.469 (9)	N5—C8	1.4628 (19)
O1S—H1S	0.87 (2)	N5—H5	0.86 (2)
C1S—C2S	1.500 (5)	N6—C7	1.334 (2)
C1S—H1SA	0.9900	N6—H6A	0.85 (2)
C1S—H1SB	0.9900	N6—H6B	0.90 (2)
C2S—H2SA	0.9800	C1—C2	1.418 (2)
C2S—H2SB	0.9800	C1—H1	0.9500
C2S—H2SC	0.9800	C2—C3	1.372 (2)
C1SA—C2SA	1.40 (2)	C2—H2	0.9500
C1SA—H1SC	0.9900	C3—C4	1.4047 (19)
C1SA—H1SD	0.9900	C3—H3	0.9500
C2SA—H2SD	0.9800	C4—C6	1.410 (2)
C2SA—H2SE	0.9800	C4—C5	1.4227 (19)
C2SA—H2SF	0.9800	C8—C10	1.517 (2)
N1—C1	1.3202 (19)	C8—C9	1.523 (2)
N1—C5	1.3651 (18)	C9—H9A	0.9800
N2—C5	1.3425 (18)	C9—H9B	0.9800
N2—N3	1.3749 (17)	C9—H9C	0.9800
N3—C6	1.3508 (17)	C10—H10A	0.9800
N3—C8	1.4682 (18)	C10—H10B	0.9800
N4—C7	1.3299 (19)	C10—H10C	0.9800
N4—C6	1.3678 (18)		
			
C1S—O1S—C1SA	36.3 (5)	N1—C1—C2	125.71 (14)
C1S—O1S—H1S	106.7 (14)	N1—C1—H1	117.1
C1SA—O1S—H1S	102.9 (14)	C2—C1—H1	117.1
O1S—C1S—C2S	110.4 (3)	C3—C2—C1	119.98 (13)
O1S—C1S—H1SA	109.6	C3—C2—H2	120.0
C2S—C1S—H1SA	109.6	C1—C2—H2	120.0
O1S—C1S—H1SB	109.6	C2—C3—C4	116.60 (13)
C2S—C1S—H1SB	109.6	C2—C3—H3	121.7
H1SA—C1S—H1SB	108.1	C4—C3—H3	121.7
C1S—C2S—H2SA	109.5	C3—C4—C6	136.46 (13)
C1S—C2S—H2SB	109.5	C3—C4—C5	119.08 (13)
H2SA—C2S—H2SB	109.5	C6—C4—C5	104.45 (12)
C1S—C2S—H2SC	109.5	N2—C5—N1	122.69 (12)
H2SA—C2S—H2SC	109.5	N2—C5—C4	113.00 (12)
H2SB—C2S—H2SC	109.5	N1—C5—C4	124.30 (13)
C2SA—C1SA—O1S	110.6 (11)	N3—C6—N4	123.49 (12)
C2SA—C1SA—H1SC	109.5	N3—C6—C4	104.98 (12)
O1S—C1SA—H1SC	109.5	N4—C6—C4	131.53 (13)
C2SA—C1SA—H1SD	109.5	N4—C7—N6	119.96 (13)
O1S—C1SA—H1SD	109.5	N4—C7—N5	122.51 (14)
H1SC—C1SA—H1SD	108.1	N6—C7—N5	117.38 (13)
C1SA—C2SA—H2SD	109.5	N5—C8—N3	104.50 (11)
C1SA—C2SA—H2SE	109.5	N5—C8—C10	108.70 (13)
H2SD—C2SA—H2SE	109.5	N3—C8—C10	110.36 (13)
C1SA—C2SA—H2SF	109.5	N5—C8—C9	111.15 (13)
H2SD—C2SA—H2SF	109.5	N3—C8—C9	109.45 (12)
H2SE—C2SA—H2SF	109.5	C10—C8—C9	112.40 (14)
C1—N1—C5	114.26 (12)	C8—C9—H9A	109.5
C5—N2—N3	102.25 (11)	C8—C9—H9B	109.5
C6—N3—N2	115.31 (11)	H9A—C9—H9B	109.5
C6—N3—C8	122.17 (12)	C8—C9—H9C	109.5
N2—N3—C8	122.23 (11)	H9A—C9—H9C	109.5
C7—N4—C6	114.85 (12)	H9B—C9—H9C	109.5
C7—N5—C8	121.41 (12)	C8—C10—H10A	109.5
C7—N5—H5	120.0 (13)	C8—C10—H10B	109.5
C8—N5—H5	111.9 (13)	H10A—C10—H10B	109.5
C7—N6—H6A	116.8 (13)	C8—C10—H10C	109.5
C7—N6—H6B	118.9 (13)	H10A—C10—H10C	109.5
H6A—N6—H6B	120.5 (18)	H10B—C10—H10C	109.5
			
C1SA—O1S—C1S—C2S	21.3 (7)	C8—N3—C6—C4	174.81 (13)
C1S—O1S—C1SA—C2SA	−39.4 (9)	C7—N4—C6—N3	−12.5 (2)
C5—N2—N3—C6	−1.02 (16)	C7—N4—C6—C4	168.49 (15)
C5—N2—N3—C8	−175.06 (13)	C3—C4—C6—N3	178.26 (17)
C5—N1—C1—C2	−2.2 (2)	C5—C4—C6—N3	−0.17 (15)
N1—C1—C2—C3	−0.2 (2)	C3—C4—C6—N4	−2.6 (3)
C1—C2—C3—C4	2.3 (2)	C5—C4—C6—N4	178.95 (15)
C2—C3—C4—C6	179.72 (16)	C6—N4—C7—N6	−173.68 (14)
C2—C3—C4—C5	−2.0 (2)	C6—N4—C7—N5	1.7 (2)
N3—N2—C5—N1	−177.97 (13)	C8—N5—C7—N4	26.4 (2)
N3—N2—C5—C4	0.87 (16)	C8—N5—C7—N6	−158.08 (14)
C1—N1—C5—N2	−178.85 (14)	C7—N5—C8—N3	−37.89 (18)
C1—N1—C5—C4	2.4 (2)	C7—N5—C8—C10	−155.73 (14)
C3—C4—C5—N2	−179.23 (13)	C7—N5—C8—C9	80.06 (17)
C6—C4—C5—N2	−0.46 (17)	C6—N3—C8—N5	27.26 (18)
C3—C4—C5—N1	−0.4 (2)	N2—N3—C8—N5	−159.11 (12)
C6—C4—C5—N1	178.35 (13)	C6—N3—C8—C10	143.94 (14)
N2—N3—C6—N4	−178.45 (13)	N2—N3—C8—C10	−42.42 (19)
C8—N3—C6—N4	−4.4 (2)	C6—N3—C8—C9	−91.86 (17)
N2—N3—C6—C4	0.77 (17)	N2—N3—C8—C9	81.78 (17)

### Hydrogen-bond geometry (Å, °)

**Table:**

*D*—H···*A*	*D*—H	H···*A*	*D*···*A*	*D*—H···*A*
N5—H5···N1^i^	0.86 (2)	2.16 (2)	3.0077 (18)	169.5 (18)
N6—H6B···N2^i^	0.90 (2)	2.08 (2)	2.9754 (18)	171.5 (18)
N6—H6A···O1S^ii^	0.85 (2)	2.03 (2)	2.8540 (18)	163.2 (18)
O1S—H1S···N4	0.87 (2)	1.93 (2)	2.7943 (17)	170 (2)

Symmetry codes: (i) −*x*+3/2, *y*−1/2, −*z*+3/2; (ii) −*x*+1, −*y*, −*z*+1.
